# Lysosomal activity maintains glycolysis and cyclin E1 expression by mediating Ad4BP/SF-1 stability for proper steroidogenic cell growth

**DOI:** 10.1038/s41598-017-00393-4

**Published:** 2017-03-21

**Authors:** Jhih-Siang Syu, Takashi Baba, Jyun-Yuan Huang, Hidesato Ogawa, Chi-Han Hsieh, Jin-Xian Hu, Ting-Yu Chen, Tzu-Chien Lin, Megumi Tsuchiya, Ken-Ichirou Morohashi, Bu-Miin Huang, Fu-l. Lu, Chia-Yih Wang

**Affiliations:** 1 Department of Cell Biology and Anatomy, College of Medicine, National Cheng Kung University, Tainan, 701 Taiwan; 2 Institute of Basic Medical Sciences, College of Medicine, National Cheng Kung University, Tainan, 701 Taiwan; 3 Division of Molecular Life Science, Graduate School of Systems Life Science, Kyushu University, Maidashi 3-1-1, Higashi-ku, Fukuoka 812-8582 Japan; 4 Department of Molecular Biology, Graduate School of Medical Sciences, Kyushu University, Maidashi 3-1-1, Higashi-ku, Fukuoka 812-8582 Japan; 5 Division of Genetics, Department of Obstetrics and Gynecology, National Cheng Kung University Hospital, Tainan, 704 Taiwan; 6 Advanced ICT Research Institute Kobe, National Institute of Information and Communications Technology, Iwaoka 588-2, Nishi-ku, Kobe 651-2492 Japan; 7 Department of Biotechnology and Bioindustry Sciences, College of Bioscience and Biotechnology, National Cheng Kung University, Tainan, 701 Taiwan; 8 Institute of Biotechnology, College of Bioscience and Biotechnology, National Cheng Kung University, Tainan, 701 Taiwan; 9 Graduate School of Frontier Biosciences, Osaka University, 1-3 Yamadaoka, Suita, 565-0871 Japan; 10 Department of Medical Research, China Medical University Hospital, China Medical University, Taichung, 404 Taiwan

## Abstract

The development and differentiation of steroidogenic organs are controlled by Ad4BP/SF-1 (adrenal 4 binding protein/steroidogenic factor 1). Besides, lysosomal activity is required for steroidogenesis and also enables adrenocortical cell to survive during stress. However, the role of lysosomal activity on steroidogenic cell growth is as yet unknown. Here, we showed that lysosomal activity maintained Ad4BP/SF-1 protein stability for proper steroidogenic cell growth. Treatment of cells with lysosomal inhibitors reduced steroidogenic cell growth *in vitro*. Suppression of autophagy did not affect cell growth indicating that autophagy was dispensable for steroidogenic cell growth. When lysosomal activity was inhibited, the protein stability of Ad4BP/SF-1 was reduced leading to reduced S phase entry. Interestingly, treatment of cells with lysosomal inhibitors reduced glycolytic gene expression and supplying the cells with pyruvate alleviated the growth defect. ChIP-sequence/ChIP studies indicated that Ad4BP/SF-1 binds to the upstream region of *Ccne1* (cyclin E1) gene during G1/S phase. In addition, treatment of zebrafish embryo with lysosomal inhibitor reduced the levels of the interrenal (adrenal) gland markers. Thus lysosomal activity maintains steroidogenic cell growth via stabilizing Ad4BP/SF-1 protein.

## Introduction

Steroid hormones, such as adrenocorticoids (glucocorticoid and mineralocorticoid), and sex steroids, are mainly produced by the adrenal cortex and gonads; the former steroids maintain energy as well as ionic homeostasis, while the latter steroids are required for sex differentiation and reproductive function^1^. All these steroids are synthesized from the common precursor cholesterol. The production of steroids, also known as steroidogenesis, is regulated by several steroidogenic enzymes^2^. Cholesterol is transported into mitochondrial inner membrane by StAR protein. In the mitochondria, pregnenolone is synthesized through side-chain cleavage of cholesterol by CYP11A1 mediating the rate-limiting step of steroidogenesis. Thereafter, the pregnenolone is catalyzed by other steroidogenic enzymes to produce kinds of steroids^3^. All these steroidogenic enzymes are mainly controlled by adrenal 4 binding protein/steroidogenic factor 1 (Ad4BP/SF-1, NR5A1)^4^.

Ad4BP/SF-1 is a tissue type-specific transcription factor belonging to nuclear receptor superfamily^5^. It is mainly expressed in the steroidogenic adrenal gland and gonads, and whereby regulate steroidogenic gene expression. In addition to the implication of Ad4BP/SF-1 into steroidogenic regulation, Ad4BP/SF-1 plays an essential role in the development of steroidogenic organs. Indeed, *Ad4BP/SF*-*1* knockout mice failed to develop the adrenal gland and gonads^6^. Although the reason why the steroidogenic organs disappeared from the KO mice was unclear, recent studies provided clues to uncover the issue. A study demonstrated that Ad4BP/SF-1 regulates the expressions of glycolytic genes, and thus providing energy for cell proliferation^7^. In addition to the function as a transcription factor, Ad4BP/SF-1 localizes to the centrosome^8^, and thus maintains centrosome configuration and homeostasis for proper mitosis and genomic integrity^9–11^. Thus, precis control of Ad4BP/SF-1 functions is required for proper steroidogenic organ development.

Lysosomes are membrane-bound organelles which contain several kinds hydrolases. With regulation of acidification, activated hydrolases degrade several substrates which derived from endocytic and autophagic pathways^12^. In the lysosomes, cholesteryl esters are hydrolyzed by a lysosomal acid lipase to produce free cholesterol for steroidogenesis. Inhibition of lysosomal activity by chloroquine reduces the degradation of cholesteryl ester to free cholesterol and resulted in decrease of low-density lipoprotein-induced progesterone production^13, 14^. In addition to releasing free cholesterol, with unknown mechanism, lysosomal activity also participates in controlling steroidogenic enzyme expressions^15^. Besides, a recent study shows that lysosomal activity enables adrenocortical cells to survive during DNA damage response^16^, however, whether lysosomal activity plays an essential role for proper steroidogenic organ development is still unclear. Here we show that lysosomal activity maintains steroidogenic cell growth *in vitro* and *in vivo* by controlling Ad4BP/SF-1 protein stability. Reduced Ad4BP/SF-1 stability leads to suppression of glycolytic genes and abnormal centrosome amplification followed by reduced S phase entry. In addition, Ad4BP/SF-1 binds to the promoter region of *Ccne1* gene thus regulating its expression during G1/S transition. These data reveal the molecular mechanism by which lysosomal activity regulates steroidogenic cell growth via controlling Ad4BP/SF-1 stability.

## Results

### Lysosomal activity maintains steroidogenic cell growth

**Figure 1:**
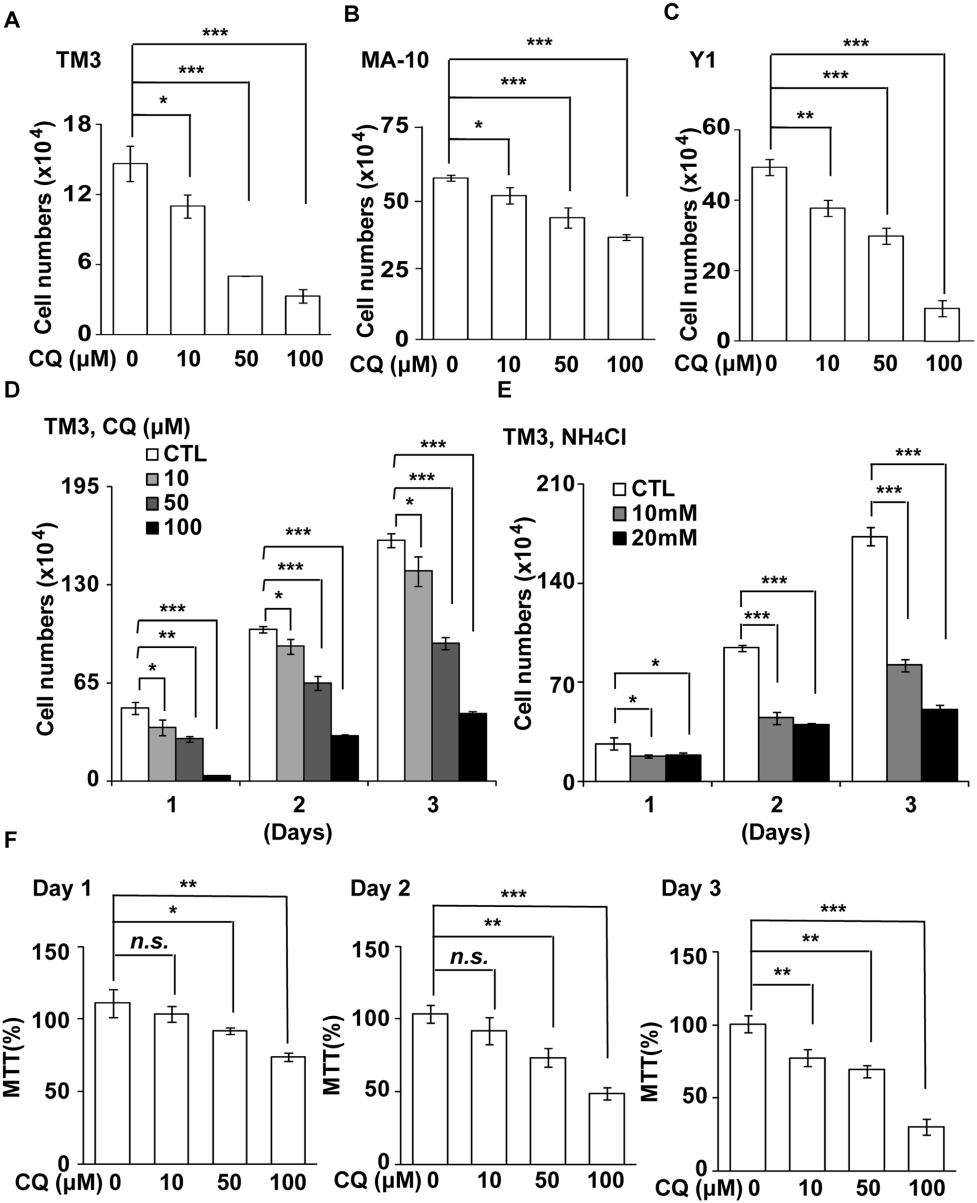
Lysosomal inhibitors reduces steroidogenic cell growth *in vitro*. (**A**–**C**) Treatment of chloroquine (CQ, at concentration of 10, 50, or 100 μM) for 24 h reduces the growth of TM3 (A), MA-10 (**B**), and Y1 (**C**) cell lines. (**D**,**E**) Treatment of TM3 cells with CQ (**D**) or NH_4_Cl (**E**) at different concentrations for different time points inhibits cell growth. CTL: control. (**F**) CQ inhibits TM3 cell growth in dose- and time- dependent manners as shown by MTT assay. n.s.: no significance; *P < 0.05; **P < 0.01; ***P < 0.001.

Lysosomal activity is required for steroidogenesis^15^. However, its role on steroidogenic cell growth is unclear. To examine it, mouse adrenocortical tumor Y1, progenitor Leydig TM3, and Leydig tumor MA-10 cells were treated with lysosomal inhibitors, chloroquine (CQ), ammonium chloride (NH_4_Cl) and bafilomycin A1 (Baf), and the growth rates were measured. When TM3 cells were treated with CQ and NH_4_Cl, LC3 (a substrate of lysosome) puncta scatted in the cytoplasm were increased (Fig. S1A). In addition, when TM3, MA-10 and Y1 cells were treated with CQ or NH_4_Cl, the amounts of LC3 and another lysosomal substrate p62 were increased in a dose-dependent manner (Fig. S1B–D). These data indicated that lysosomal activities were blocked efficiently by these reagents. The effect of the lysosomal inhibitors on steroidogenic cell growth was further examined. By counting cell numbers and performing MTT assay, we found that CQ, NH_4_Cl, and Baf reduced a number of all cell lines tested dose- and time-dependently (Fig. 1A–F and S2A). In addition, CQ hardly induced cell death (Fig. S2C and F). Thus, pharmacological inhibition of lysosomes suppressed steroidogenic cell growth *in vitro*.

**Figure 2:**
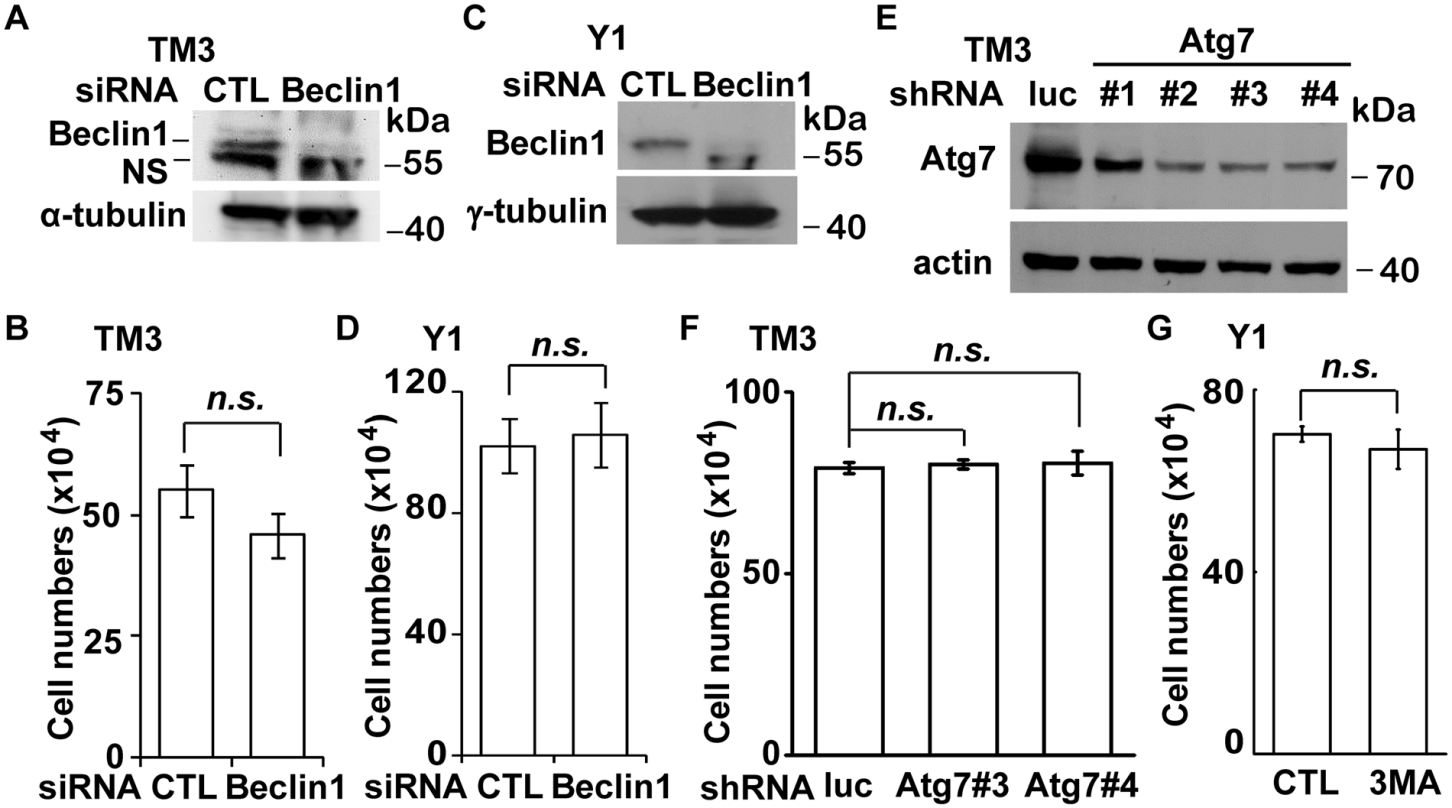
Autophagy is dispensability for steroidogenic cell growth. (**A**–**D**) Depletion of Beclin 1 does not affect steroidogenic cell growth. (**A** and **C**) Whole cell extracts of siRNA against Beclin1-treated TM3 (**A**) or Y1 (**C**) cell lines were analyzed by immunoblot with antibodies against Beclin1, α-tubulin, and γ-tubulin. NS: non-specific signal. (**B** and **D**) Quantitation of cell numbers of Beclin1 depleted TM3 (**B**) or Y1 (**D**) cell lines. (**E**,**F**) Depletion of Atg7 does not affect TM3 cell growth. (**E**) Whole cell extracts of lentivirus containing shRNA against Atg7 (shAtg7#1-4)-infected TM3 cell line were analyzed by immunoblot with antibodies against Atg7 and actin. (**F**) Quantitation of cell numbers of Atg7 depleted TM3 cell line. (**G**) Treatment of cells with autophagic inhibitor 3MA (2 mM) does not affect cell growth. Quantitation of cell numbers of 3MA-treated Y1 cells. These results are mean +/− SD from three independent experiments. n.s.: no significance.

Autophagy is a lysosomal-degradation process whereby cells degrade and reutilize old organelles and proteins to maintain metabolic homeostasis^17^. Since inhibition of lysosomes blocks autophagic flux^18^, the effect of autophagy on steroidogenic cell growth was examined. Beclin1 encoded by *Becn 1* is a critical factor for autophagy initiation. Depletion of Beclin1 by siRNA did not affect the growth of TM3 and Y1 cells (Fig. 2A–D). To further confirm this, the expression of *Atg7*, which encodes an E1-like activating enzyme that is essential for autophagy, was inhibited by infected cells with lentivirus-containing shRNA against *Atg7*. Four shRNA sequences were analyzed and found the shAtg7#3 and shAtg7#4 inhibited Atg7 expression efficiently (Fig. 2E). Then we tested whether depletion of Atg7 affected steroidogenic cell growth. In consistent with the result obtained from beclin1 deficient cells, depletion of Atg7 did not affect steroidogenic cells growth (Fig. 2F). These were further confirmed by treating cells with 3-Methyladenine (3-MA), an autophagic inhibitor blocks autophagosome formation. In the presence of 3-MA at 2 mM concentration, Y1 cell growth was not affected (Fig. 2G and S2B). However, higher concentration of 3-MA treatment (5 or 10 mM) induced cell death, this result is consistent with previous study that 3-MA inhibits autophagy at lower concentration but induces cellular apoptosis at higher concentration (5 or 10 mM)^19^. Collectively, these data indicates that the steroidogenic cell growth reduced by lysosome inhibitors was not due to deficient autophagic flux. This result is consistent with recent studies showing that ablation of Beclin 1 did not affect the growth of steroidogenic granulosa and luteal cells in the ovary, even though the steroidogenesis is inhibited^20, 21^. Taken together, autophagy seems to be dispensable for steroidogenic cell growth.

### Pharmacological inhibition of lysosomes leads to reduced S phase entry in steroidogenic cells

**Figure 3:**
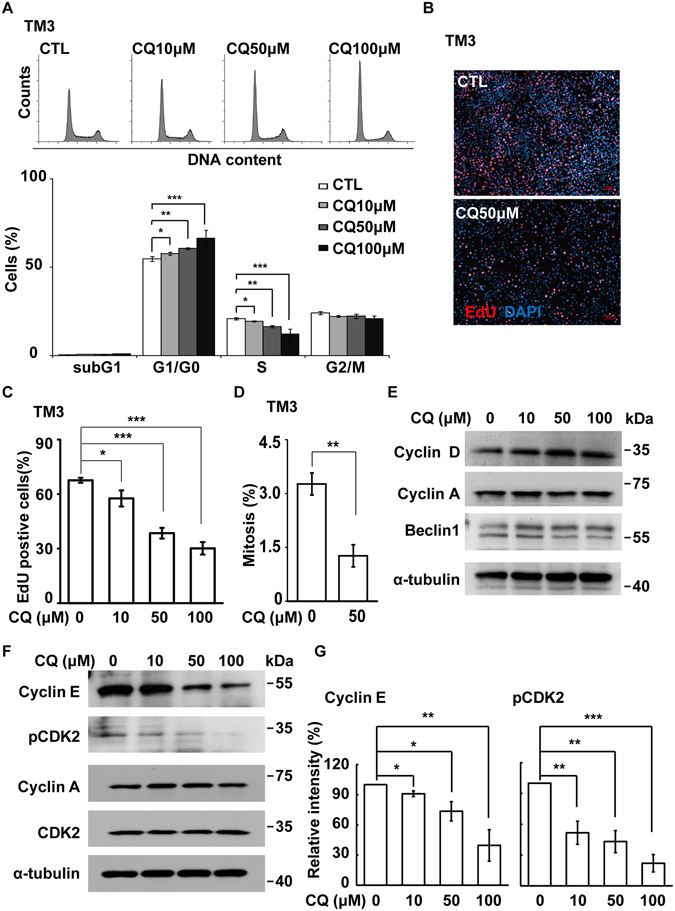
Inhibition of lysosomes leads to G1 arrest by inactivating cyclin E/CDK2 complex. (**A**) CQ treatment (10, 50, or 100 μM, 24 h) leads to cell cycle G1 arrest. Quantitation of different cell cycle stages in TM3 cells in the presence or absence of CQ. (**B**–**D**) EdU incorporation and mitotic index are reduced in CQ-treated TM3 cells. (**B**) Immunostaining of EdU (red) and DAPI (blue) in scramble control (CTL) or CQ treated TM3 cells. Quantitation of EdU incorporation (**C**) or mitotic index (**D**) in scramble control (CTL) or CQ-treated TM3 cells. These results are mean +/− SD from three independent experiments; more than 1000 cells were counted in each individual group. (**E**–**G**) CQ inhibited cyclin E1 expression and CDK2 activation. (**E**,**F**) Whole cell extracts of CQ-treated TM3 cells at the concentration of 10, 50, or 100 μM are analyzed by immunoblot with antibodies against Beclin1, cyclin D, cyclin A, cyclin E1, CDK2, phosphorylated CDK2 at Thr160 (pCDK2) and α-tubulin. (**G**) Quantitation of relative intensity of cyclin E and pCDK2 in (**F**). *P < 0.05; **P < 0.01; ***P < 0.001.

Since cell growth was affected by the treatment of lysosomal inhibitors, we analyzed the cell cycle profile. When cells were treated with CQ, the population of cells at G1 phase was increased whereas that at S phase was reduced (Fig. 3A and S2C). The ability of cells to enter into S phase was studied by EdU incorporation assay. Consistent with our flow cytometry analysis, inhibition of lysosomes reduced EdU incorporation dose-dependently in TM3 cells (Fig. 3B,C). Similar effect of lysosomal inhibition was observed in MA-10 and Y1 cells (Fig. S2D,E). Then we checked the ability of cells entered M phase by examining mitotic index. The mitotic index was reduced (Fig. 3D) when the cells were treated with lysosomal inhibitor. We further checked whether inhibition of lysosomes induced cell death. Treatment with CQ at low doses (10 and 50 μM) did not induce cell death (Fig. 3A, S2C and S2F), while treatment with higher concentration of CQ (100 μM) modestly induced cell death as shown by an increase of cleaved caspase 3 in Y1 cells (Fig. S2G). Thus the ability of cells enters S phase is reduced in the presence of lysosomal inhibitors.

Since cyclin and CDK complexes are important for cell cycle progression^22^, we checked the expression of cyclins and the activation of CDK2. CKD2 has been known to be active when Thr160 is phosphorylated (pCDK2). The expression levels of cyclin D, cyclin A, and CDK2 were not affected when cells were treated with CQ. However, the amounts of cyclin E1 and pCDK2, which are crucial for G1/S transition^23, 24^, were reduced by the treatment (Fig. 3E–G). Reduced EdU incorporation and cyclin E/CDK2 activation supported the notion that lysosomal activity is required for G1/S transition in steroidogenic cell growth.

### Lysosomal activity maintains steroidogenic cell growth by controlling Ad4BP/SF-1

**Figure 4:**
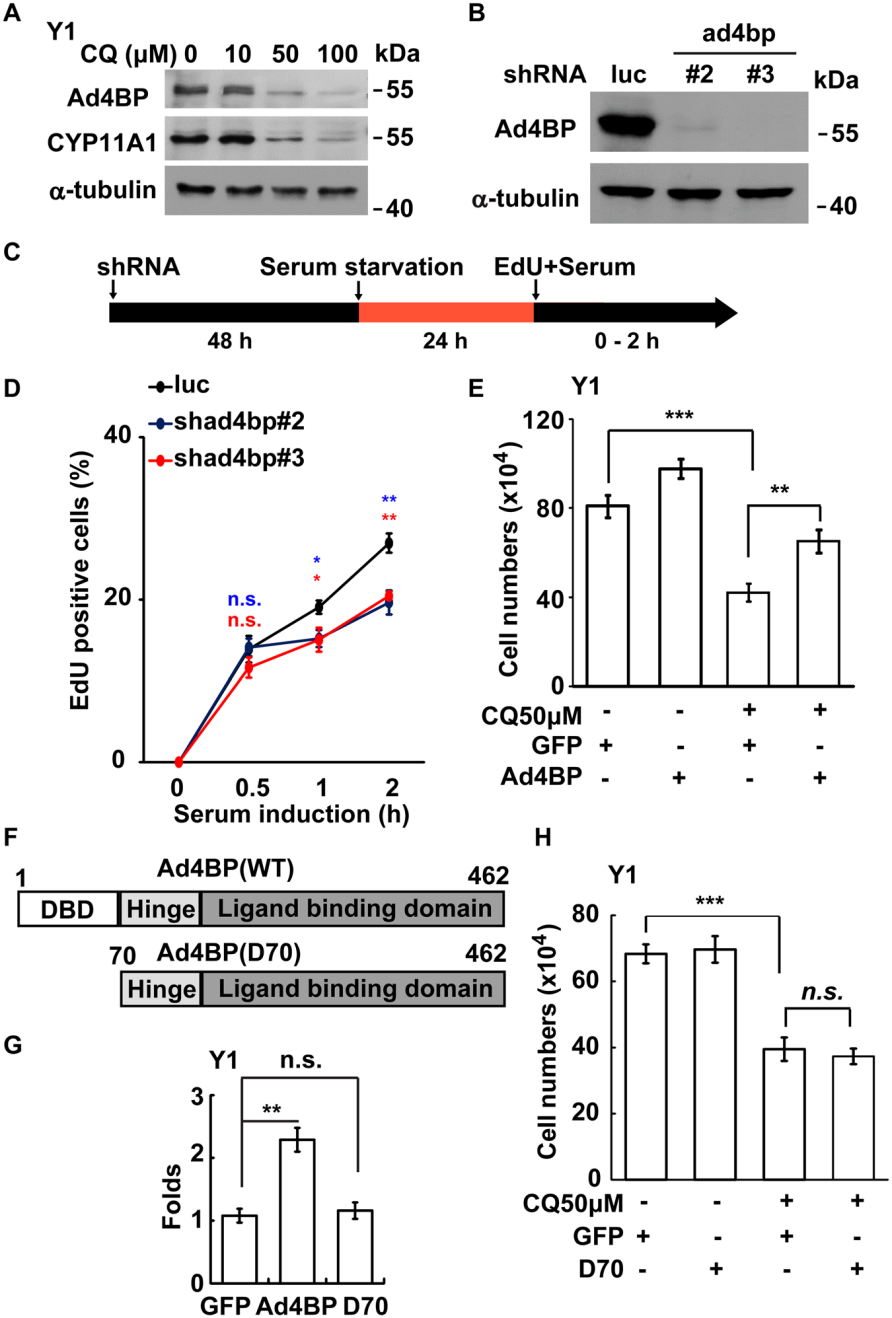
Lysosomal activity maintains the abundance of Ad4BP/SF-1 for steroidogenic cell growth. (**A**) Inhibition of lysosomes reduces the level of Ad4BP/SF-1. Whole cell extracts of CQ-treated TM3 cells at the concentration of 10, 50, or 100 μM ware analyzed by immunoblot with antibodies against Ad4BP/SF-1(Ad4BP), CYP11A1 or actin. (**B**–**D**) Depletion of Ad4BP/SF-1 prevents S phase entry. (**B**) Ad4BP/SF-1 is depleted by shRNA efficiently. Whole cell extracts of lentivirus containing shRNA against Ad4BP/SF-1 (#2 and #3)-infected Y1 cells are analyzed by immunoblot with antibodies against Ad4BP/SF-1 and α-tubulin. (**C**) The diagram of experimental procedure. Cells were infected with lentivirus and incubated for two days followed by 24 h serum starvation. Before harvesting, the culture medium was added with serum and EdU. (**D**) Percentage of EdU-incorporating cells after infection with control shRNA (shluc) or shRNA against Ad4BP (shad4bp#2 and #3). Values are calculated as the percentages of cells positive for EdU, whose total number within each experiment was assumed to be 100%. (**E**) Overexpression of Ad4BP/SF-1 rescues CQ-induced growth delay. Quantitation of GFP or Ad4BP/SF-1 overexpressed Y1 cell numbers in the presence or absence of CQ (50 μM). (**F**,**H**) Transcriptional activity is required for cell growth in CQ-treated Y1 cells. (**F**) Schematic representation of the experimental construct of wild-type (WT) or DNA-binding domain-deleted (D70) Ad4BP/SF-1. DBD: DNA-binding domain; Hinge: hinge domain. (**G**) The Luciferase activity of Ad4BP/SF-1 dependent reporter *phscc*-*2*.*3K*-*Luc* were determined after co-transfection with expression plasmid of GFP, wild-type (WT) Ad4BP/SF-1, or DNA-binding deficient (D70) Ad4BP/SF-1 in Y1 cells. (**H**) Overexpression of DNA-binding deficient Ad4BP/SF-1 dose not rescue cell growth in CQ-treated Y1 cells. Quantitation of GFP or D70 overexpressed Y1 cell numbers in the presence or absence of CQ (50 μM). n.s.: no significance; *P < 0.05; **P < 0.01; ***P < 0.001.

Since Ad4BP/SF-1 is required for proper growth of steroidogenic organs such as adrenal gland and gonads^6^, its expression was examined in the cells treated with lysosomal inhibitor. The amount of Ad4BP/SF-1 in TM3, Y1, and MA-10 cells treated with CQ was reduced in a dose-dependent manner (Fig. 4A and S3A–C). Similar effect was observed by an NH_4_Cl treatment (Fig. S3D). Consistent with it, the expression of downstream target genes of Ad4BP/SF-1, *Star* and *Cyp11a1*, were decreased in CQ-treated Y1 and TM3 cells (Fig. 4A and S2E–G). These data indicate that Ad4BP/SF-1-regulated steroidogenic genes are downregulated by lysosomal inhibitors.

To further investigate whether Ad4BP/SF-1 is required for G1/S transition, the EdU incorporation assay was performed with the cells in which Ad4BP/SF-1 was depleted by shRNAs. The expression of Ad4BP/SF-1 was reduced efficiently by shRNA-containing lentivirus infection (Fig. 4B). The number of cells to enter into S phase was reduced by the treatment (Fig. 4C,D), indicating that Ad4BP/SF-1 is required for G1/S transition. To confirm it, wild-type (WT) Ad4BP/SF-1 or GFP were overexpressed in CQ-treated Y1 cells. As the consequence, we found that the overexpression of Ad4BP/SF-1, but not GFP, rescued cell growth affected by CQ treatment (Fig. 4E). To examine whether the transcriptional activity of Ad4BP/SF-1 is required for the rescue, the DNA-binding domain-depleted Ad4BP/SF-1 (D70) was used. This D70 truncated form was transcriptionally inactive (Fig. 4F,G), and its overexpression did not rescue the CQ-induced growth defect (Fig. 4H). These results strongly suggests that Ad4BP/SF-1 regulates G1/S transition possibly through transcription activity.

Since Ad4BP/SF-1 maintains steroidogenic cell growth and proper mitotic entry by controlling centriole homeostasis^8, 10^ and inhibition of lysosome also leads to mitotic delay, we then examined the numbers of centriole. During interphase, untreated cells contained 2 (unduplicated, G1 phase) or 4 (duplicated, S/G2 phase) centrioles. Upon CQ-treated, the population of abnormal cells with multiple centrioles (>4 centrioles) was increased in TM3 cells (Fig. S4A,B), indicating that Ad4BP/SF-1-controlled centriole homeostasis was disturbed.

**Figure 5:**
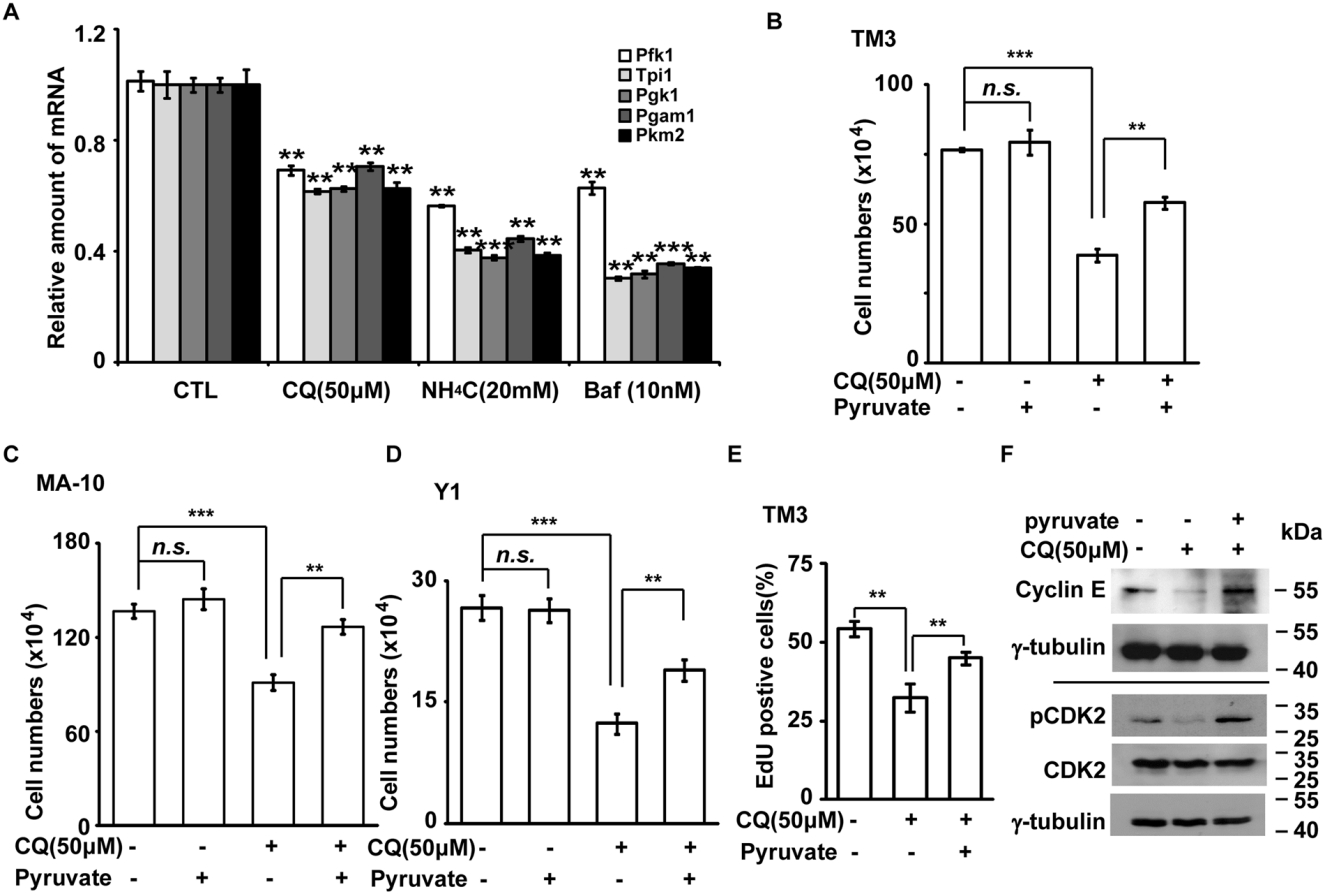
Glycolytic gene expression is reduced by treating cells with lysosomal inhibitors. (**A**) Glycolytic gene expression is reduced by treating cells with lysosomal inhibitors. Quantitation of glycolytic gene expression in lysosomal inhibitors-treated Y1 cells (CQ: 50 μM; NH_4_Cl: 20 mM; Baf: 10 nM). CTL: control; CQ: chloroquine; Baf: bafilomycin A1. (B-D) Pyruvate rescues cell growth in CQ (50 μM)-treated TM3 (**B**), MA-10 (**C**), and Y1 (**D**) cell lines. (**E**) Pyruvate promotes S phase entry in CQ (50 μM)-treated TM3 cells. (**F**) Pyruvate restores cyclin E1 expression and CDK2 activation. Whole cell extracts of Y1 cells treated with or without CQ (50 μM) in the presence or absence of pyruvate are analyzed by immunoblot with antibodies against cyclin E1, phosphorylated CDK2 at Thr160 (pCDK2) and γ-tubulin. n.s.: no significance; *P < 0.05; **P < 0.01; ***P < 0.001.

In addition to controlling centriole homeostasis, Ad4BP/SF-1 regulates glycolytic genes, and thereby providing energy sufficient for cell cycle progression^7^. Glycolytic gene expression was examined in the cells treated with lysosomal inhibitors. As expected, treatment of cells with CQ, NH_4_Cl, and Baf reduced the expression of glycolytic genes, such as *Pfk1*, *Tpi1*, *Pgk1*, *Pgam1*, and *Pkm2*, suggesting that Ad4BP/SF-1-regulated glycolysis was impaired by lysosome inhibition (Fig. 5A). Interestingly, it was shown that Ad4BP/SF-1 knockdown led to drastic reduction of the amount of a glycolytic end-product, pyruvate^7^. Then, we checked whether pyruvate supplementation could rescue the growth defect induced by lysosomal inhibitors. Addition of pyruvate ameliorated the growth defect of TM3, Y1, and MA-10 cells induced by the CQ treatment (Fig. 5B–D). Likewise, EdU incorporation and the expression of cyclin E and pCDK2 were restored by the pyruvate supplementation to the CQ treated cells (Fig. 5E,F). Thus, our data suggests that lysosome inhibition down-regulates Ad4BP/SF-1 expression followed by inducing abnormal centriole amplification and reducing glycolysis.

**Figure 6:**
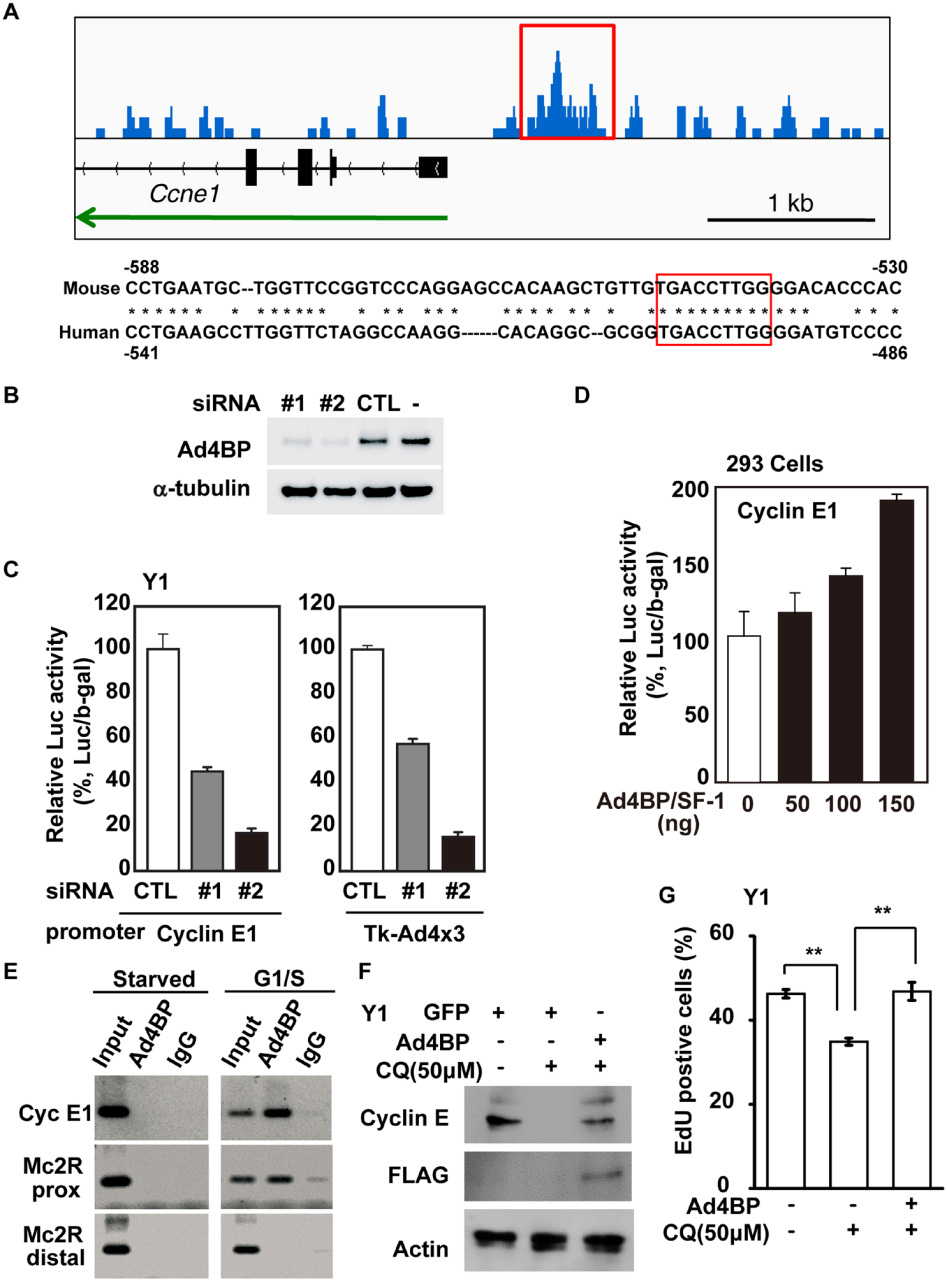
Ad4BP/SF-1 binds to the promoter of *Ccne1*. (**A**) Visualization of the accumulation of Ad4BP/SF-1 at promoter region of *Ccne1* gene (upper). A green arrow shows the direction of *Ccne1* gene. Nucleotide sequences of the ChIP-peak regions are compared between mouse and human (bottom). A red box indicates conserved binding sequence for Ad4BP/SF-1. (**B**) Ad4BP/SF-1 is depleted by siRNA efficiently. Whole cell extracts of siRNA against Ad4BP/SF-1 (#1 and #2) transfected Y1 cells are analyzed by immunoblot with antibodies against Ad4BP/SF-1 and α-tubulin. (**C**) A *Ccne1*-Luc was transfected into Y1 cells with siRNAs against Ad4BP/SF-1 (#1 and #2). Average values and SDs of the luciferase activities are indicated. The relative luciferase activity is shown: the amount of Ad4BP/SF-1 activation in the presence of control siRNA set at 100%. n = 3. Tk-Ad4 × 3-Luc was used as a positive control. (**D**) *Ccne1*-Luc were transfected into HEK293 cells and with increasing amounts of the expression vector for Ad4BP/SF-1. The relative luciferase activity is shown: the amount of Ad4BP/SF-1 activation in the absence of expression plasmid of Ad4BP/SF-1 set at 100%. n = 3. (**E**) Chromatin was prepared from Y-1 cells either at G0 or G1/S phase. The binding of Ad4BP/SF-1 to *Ccne1* promoter was examined by ChIP. The proximal and distal upstream regions of Mc2R gene were used as positive and negative control, respectively. The normal rabbit IgG was used as a negative control for immunoprecipitation. (**F**,**G**) Overexpression of Ad4BP/SF-1 restores cyclin E1 expression (**F**) and S phase entry (**G**) in CQ (50 μM)-treated Y1 cells. Whole cell extracts of GFP or Ad4BP/SF-1 overexpressed Y1 cells in the presence or absence of CQ (50 μM) were analyzed by immunoblot with antibodies against cyclin E1, FLAG, and actin. (**G**) Quantitation of EdU incorporation in Ad4BP/SF-1 overexpressed Y1 cells in the presence or absence of CQ (50 μM). **P < 0.01.

We then re-analyzed our previously published ChIP-sequence data^7^ and found that Ad4BP/SF-1 binds to an upstream region of *Ccne1* gene. Within the ChIP-peak region at the *Ccne1* gene upstream, we found a consensus Ad4BP/SF-1-binding sequence conserved between mouse and human (Fig. 6A). As these observations strongly suggested that Ad4BP/SF-1 directly regulated *Ccne1* gene, we performed luciferase reporter assays by using reporter gene harboring *Ccne1* gene promoter^25^. As the results, the reporter activity was decreased by the knockdown of *Ad4BP/SF*-*1* (Fig. 6B,C), while increased by its overexpression (Fig. 6D). These results indicate that *Ccne1* gene expression is, at least in part, controlled by Ad4BP/SF-1. Next, we tested whether Ad4BP/SF-1 bound to *Ccne1* promoter in a cell cycle-dependent manner. When the cells are arrested in G0 phase by serum starvation, Ad4BP/SF-1 did not bind to the *Ccne1* promoter (Fig. 6E). Interestingly, the binding appeared during G1/S phase transition, suggesting that Ad4BP/SF-1 plays a critical role for G1/S phase transition by regulating *Ccne1* gene. To further confirm this, Ad4BP/SF-1 was overexpressed in CQ-treated cells. Inhibition of lysosomes reduced the expression of Cyclin E1, and its expression was restored by Ad4BP/SF-1 overexpression (Fig. 6F). Expectedly, the enforced expression of Ad4BP/SF-1 increased S phase entry of the CQ-treated cells (Fig. 6G). Thus Ad4BP/SF-1 directly regulates the expression of Cyclin E1 for proper G1/S transition.

**Figure 7:**
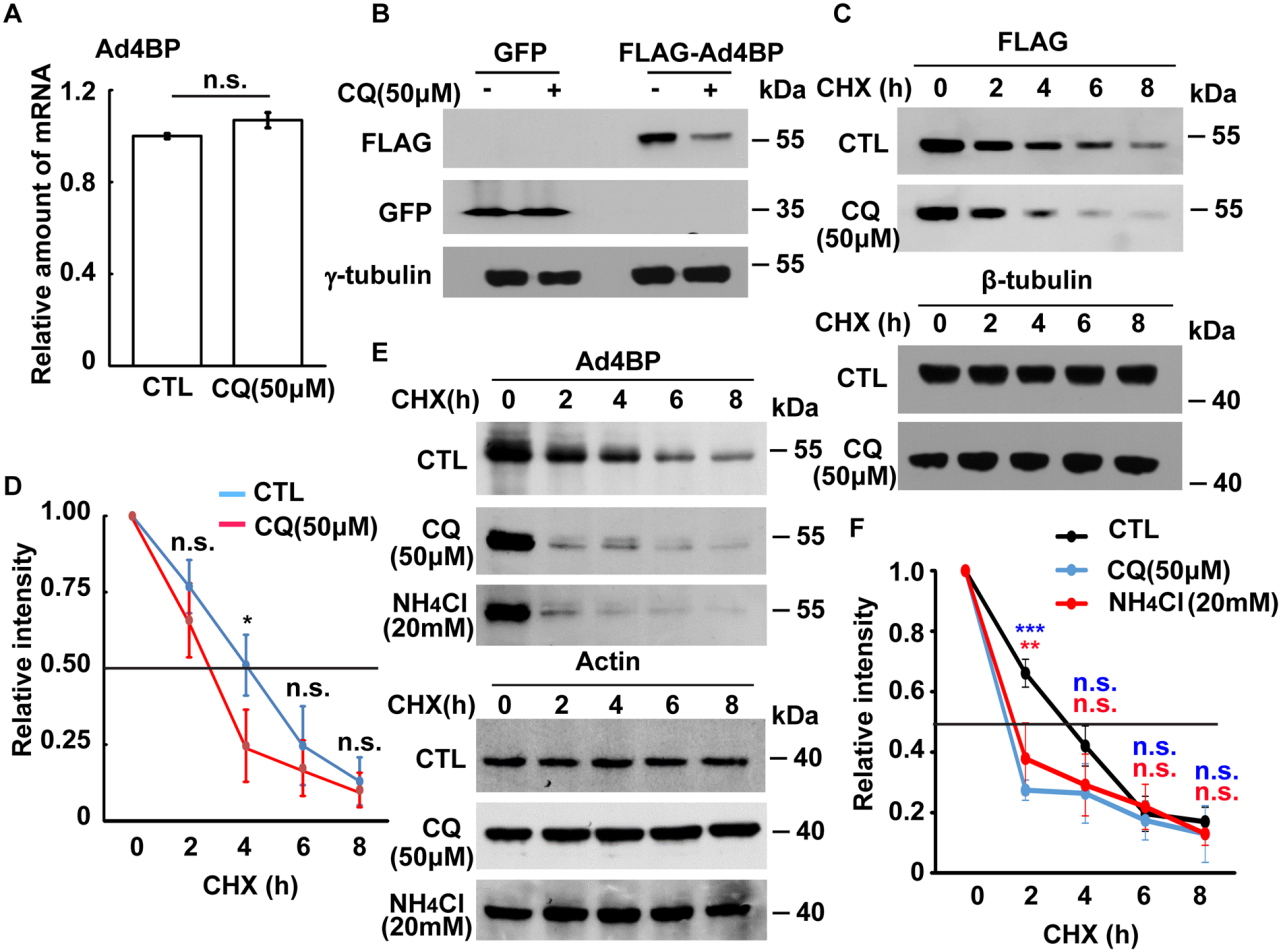
Protein stability of Ad4BP/SF-1 is reduced in CQ-treated Y1 cells. (**A**) CQ treatment does not affect mRNA level of Ad4BP/SF-1. Quantitation of mRNA level of Ad4BP/SF-1 in the presence or absence of CQ (50 μM). (**B**) Ectopic expressed Ad4BP/SF-1 is reduced in CQ-treated Y1 cells. Whole cell extracts of GFP or 3xFLAG-Ad4BP/SF-1 overexpressed Y1 cells in the presence or absence of CQ (50 μM) were analyzed by immunoblot with antibodies against FLAG, GFP, and γ-tubulin. (**C**–**F**) Protein stability of exogenous or endogenous Ad4BP/SF-1 are reduced in CQ-treated Y1 cells. (**C**) Whole cell extracts of 3xFLAG-Ad4BP/SF-1 overexpressed Y1 cells with or without cycloheximide in the presence or absence of CQ (50 μM) were analyzed by immunoblot with antibodies against FLAG and β-tubulin. (**D**) Quantitation of relative intensity of 3xFLAG-Ad4BP/SF-1 in (**C**); n.s.: no significance; *P < 0.05. (**E**) Whole cell extracts of Y1 cells treated with or without cycloheximide in the presence or absence of CQ (50 μM) or NH_4_Cl (20 mM) were analyzed by immunoblot with antibodies against Ad4BP/SF-1 (Ad4BP) and actin. (**F**) Quantitation of relative intensity of endogenous Ad4BP/SF-1 in (**E**); n.s.: no significance; **P < 0.01; ***P < 0.001.

Next we investigated how lysosomal activity affected Ad4BP/SF-1 expression. When lysosomal activity was inhibited, the mRNA level of Ad4BP/SF-1 was not affected (Fig. 7A). Nevertheless, lysosome inhibition reduced Ad4BP/SF-1 protein level, suggesting that Ad4BP/SF-1 protein stability might be affected. To confirm the possibility, 3xFLAG-tagged Ad4BP/SF-1 was expressed in Y1 cells. The ectopically expressed Ad4BP/SF-1 was largely reduced by CQ treatment (Fig. 7B). Moreover, the cycloheximide chase assay was performed. In the presence of CQ, exogenous Ad4BP/SF-1 was degraded faster than that of control treatment (Fig. 7C,D, the half-life of exogenous Ad4BP/SF-1 was 3.9 h; however, it was reduced to 2.3 h when treated with CQ). In addition, this was also observed in the endogenous Ad4BP/SF-1 when cells were treated with CQ or NH_4_Cl (Fig. 7E,F, the half-life of endogenous Ad4BP/SF-1 was 3.6 h; however, it was reduced to 2.0 h and 1.9 h when treated with CQ or NH_4_Cl, respectively). Thus, inhibition of lysosomes decreases Ad4BP/SF-1 protein stability.

**Figure 8:**
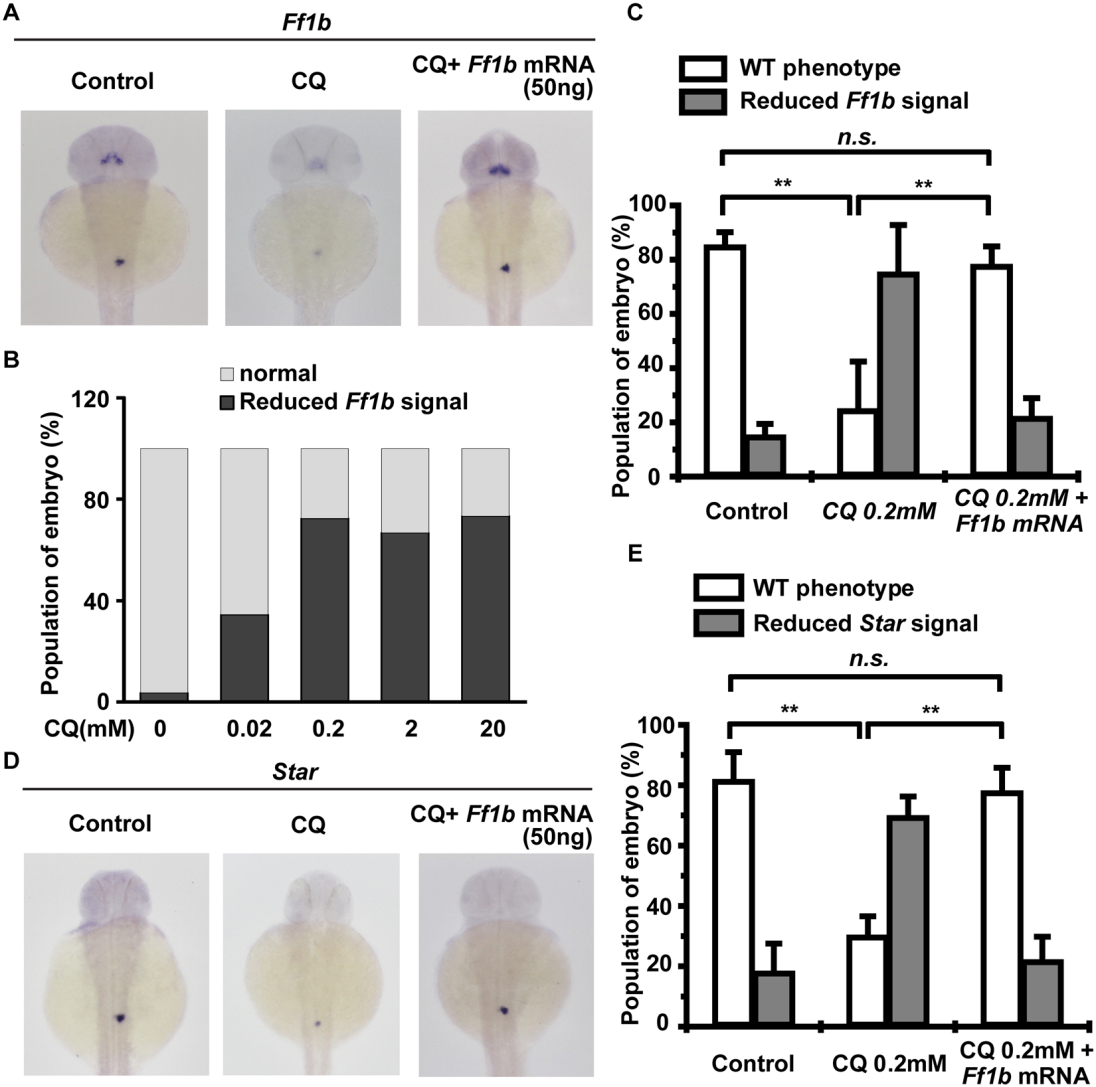
Chloroquine inhibits interrenal gland development. (**A**–**C**) Chloroquine reduces interrenal gland development in zebrafish embryo. (**A**) The whole mount *in situ* hybridization of *Ff1b* in the wild-type like (WT), or CQ-treated (reduced) with or without *Ff1b* mRNA injected zebrafish embryo. (B-C) Quantitation result of zebrafish embryo with reduced Ff1b signal in the presence of different dose of CQ (**B**) or injected with *Ff1b* mRNA (**C**). (**D**,**E**) Reduced steroidogenic enzyme expression in CQ-treated zebrafish embryo. (**D**) The whole mount *in situ* hybridization of *Star* in the wild-type like (WT) or CQ-treated (reduced) with or without *Ff1b* mRNA injected zebrafish embryo. (**E**) Quantitation result of (**D**). n.s.: no significance; **P < 0.01.

Next we checked the effect of lysosomal activity on the development of the adrenal gland (interrenal gland) in zebrafish *in vivo*. Ff1b, the zebrafish homologue of Ad4BP/SF-1, is required for the development of steroidogenic component of the zebrafish interrenal gland^26^. Treatment of zebrafish embryo with CQ inhibited the growth of the interrenal gland dose-dependently as shown by reduced *Ff1b* staining (Fig. 8A–C). In addition, the expression of a steroidogenic gene, *Star*, was also reduced (Fig. 8D,E), indicating that lysosomal activity is required for adrenal gland development in zebrafish. To further confirm the role of Ff1b in this phenotype, *Ff1b* mRNA was injected into the zebrafish embryo in the presence of CQ and found the growth of interrenal gland was rescued (Fig. 8C and E). Taken together, inhibition of lysosomal activity reduces the levels of *Ff1b* and *Star*, the markers of interrenal gland.

## Discussion

In this study, we demonstrated that lysosomal activity is relevant to steroidogenesis and steroidogenic organ development by the maintenance of Ad4BP/SF-1 protein stability (Fig. S5). Ad4BP/SF-1 is critical for adrenal gland and gonads development^6^. Here, we show that, upon treatment of lysosomal inhibitors, the protein stability of Ad4BP/SF-1 was reduced, thus leading to lower glycolysis and abnormal centrosome amplification followed by G1 arrest and reduced mitosis entry. Furthermore, we found that the expression of cyclin E1 encoded by *Ccne1* gene was directly regulated by Ad4BP/SF-1. Ad4BP/SF-1 bound to the promoter region of *Ccne1* gene thus regulating its expression during G1/S transition. The expression of cyclin E at G1/S transition is required for initiation of DNA replication^27^. In addition, higher ATP production is known to boost cyclin E level for S phase entry^28^. Taken together, through regulation of ATP production by controlling glycolysis and cyclin E1 expression during G1/S transition, Ad4BP/SF-1 seems to cooperate these two biological events at the level of gene expression.

In our cell model, we found that the mRNA level of Ad4BP/SF-1 was not affected whereas the protein stability was reduced when lysosome was inhibited. However, by using *in situ* hybridization, we found that the expression of Ff1b, the homology of Ad4BP/SF-1 in the mammalian, in the zebrafish was reduced when treated with CQ. Due to the limitation of available antibody, so far detection of the mRNA of Ff1b or its downstream genes, such as Cyp11a1 or Star, is the best method to observe the development of zebrafish adrenal gland. As we only observed the protein stability, but not mRNA, of Ad4BP/SF-1 was reduced in the cell model, we speculate that in the zebrafish model, at least in part, the reduced Ff1b and Star signals represent as reduced cell number.

Lysosome is the degradation center for the maintenance of intra-cellular homeostasis. In addition, it also contributes to the cell cycle progression. In the budding yeast, *Saccharomyces cerevisiae*, the vacuole/lysosome is required for proper growth; loss of functional vacuole leads to G1 phase arrest^29^. Here we provide further evidence to support the importance of lysosomal activity in maintaining cell cycle progression. In the steroidogenic cells, lysosomal activity maintains the protein level of Ad4BP/SF-1 thus inducing glycolysis and cyclin E1 expression for proper S phase entry. We have also tested the effect of lysosomal inhibitor on the growth of non-steroidogenic cells. As expected, the non-steroidogenic cells also show reduced S phase entery when treated with lysosomal inhibitors (data not shown). As Ad4BP/SF-1 is a tissue-type specific transcription factor, it reasons to speculate that there might be a nuclear receptor involved in the function similar to that of Ad4BP/SF-1 in non-steroidogenic cells. The estrogen-related receptor alpha (ERRα, NR3A1) is one of the candidates. It is expressed in several tissues and regulates genes involved in glucose metabolism and fatty acid oxidation^30^. In addition, ERRα binds to the same nucleotide sequence as Ad4BP/SF-1 to activate transcription^7^. Thus, it might be interesting to examine, in those non-steroidogenic cells, whether the level of the ERRα is affected, leading to reduced glycolysis followed by reduced S phase entry upon lysosomal inhibition.

Enhanced expression of Ad4BP/SF-1 has been observed frequently in steroidogenic tumors^31^. Here we show that inhibition of lysosome suppresses Ad4BP/SF-1 stability thus reducing cell cycle progression. When adrenocortical tumor cells are treated with sub-lethal dose of anti-cancer drug, chloroquine can promote apoptosis thus these tumors are less susceptible to malignancy^16^. Chloroquine is an anti-malarial drug by inhibiting lysosomal acidification in clinics^32^. Thus, we consider that lysosomal inhibitor can be used in combination with other anti-cancer drugs such as etoposide in the treatment of steroidogenic tumors; it can either reduce the amount of Ad4BP/SF-1 or improve the cytotoxicity of anti-cancer drugs thus getting better therapeutic effects.

Although the protein stability of several nuclear receptors or its transcriptional co-regulators, such as androgen receptor or androgen receptor-interacting protein 4, are regulated by autophagy during stress responses^33, 34^, whether Ad4BP/SF-1 stability is also regulated by this pathway is as yet unclear. In this study we found that the amount of Ad4BP/SF-1 was not affected by inhibition of autophagy but was reduced by lysosomal inhibition. It is known that the protein stability of Ad4BP/SF-1 is regulated by proteasome degradation pathway with Skp1/Cul1/F-box protein (SCF) family^35^. In addition, some E3 ligase, such as RING finger protein 152 (RNF152), localize to lysosome, implying that lysosomal activity might regulate E3 ligase activity^36^. Thus we speculated that when lysosomal activity is inhibited, the E3 ligase regulating Ad4BP/SF-1 stability might be activated thus leading to downregulation of Ad4BP/SF-1 via proteasome degradation pathway, but this hypothesis still need to be tested.

Previous study shows that treatment of ovarian granulosa cells with lysosomal inhibitor, chloroquine, reduces steroidogenic gene expressions^15^. However, the underlying molecular mechanism was unknown. In this study, we found that inhibition of lysosomal activity suppressed the expression of steroidogenic genes by down-regulating Ad4BP/SF-1. Ad4BP/SF-1 is the master regulator in controlling steroidogenesis^37^, besides, autophagy may also contribute to this process. In the ovary of *Becn 1* conditional knockout mice, the growth and differentiation of granulosa and luteal cells are normal. However, these mice suffered from lowered progesterone production due to less lipid droplets accumulation, thus leading to preterm labor. As lysosomal activity regulates autophagic flux, thus we consider that lysosomal activity contributes to steroidogenesis in multiple pathways, including regulating autophagy for lipid droplets accumulation, promoting free cholesterol catalysis, and, more importantly, maintaining Ad4BP/SF-1 protein stability. Once the lysosomal activity is inhibited, the autophagic flux is reduced leading to less lipid droplet accumulation; the source of free cholesterol is reduced; and the amount of Ad4BP/SF-1 is also reduced. Thus the steroidogenic pathway is disturbed leading to lower steroid production.

In summary, here we uncovered the novel function of lysosome in controlling steroidogenic gene expression and maintaining the steroidogenic cell growth by regulating Ad4BP/SF-1 protein stability.

## Methods

### Cell culture and Drug treatment

Mouse adrenocortical Y1 and mouse progenitor Leydig TM-3 and Leydig MA-10 cell lines were grown in Dulbecco’s modified Eagle medium (DMEM)-F12 medium supplemented with 10% fetal bovine serum at 37 °C in a humidified atmosphere at 5% CO2. These cells were regularly examined for the presence of Ad4BP/SF-1and free of mycoplasma contamination by immunoblot, immunofluorescence, and DAPI staining according to the guidelines. HEK 293 cells were grown in DMEM supplemented with 10% fetal bovine serum and 1x penicillin-streptomycin-glutamine at 5% CO2 and 37 °C. For drug treatment, cells were incubated with or without 10, 50, or 100 μM chloroquine (Novus Biologicals, Littleton, CO, USA), 10 or 20 mM ammonium chloride (Sigma, St. Louis, MO, USA), 1, 5, or 10 nM bafilomycin A1(Enzo Life Sciences, Farmingdale, NY, USA), 2, 5, or 10 mM 3-methyladenine (Sigma, St. Louis, MO, USA), and 1 mM sodium pyruvate (Caisson Laboratories, Logan, UT, USA) for 16 or 24 h before analysis. 50 μg/ml cycloheximide(Sigma, St. Louis, MO, USA) for 2, 4, 6, or 8 h before analysis; 20 ng/ml platelet-derived growth factor alpha polypeptide (Cell Guidance Systems, Cambridge, UK) for 24 h before analysis.

### Zebrafish maintenance and drug treatment

The Zebrafish (AB) was obtained from Taiwan Zebrafish Core Facility and maintained following the condition of zebrafish care as described in zebrafish book (ZFIN). The experimental procedures were performed under the Institutional Animal Care and Use protocol from National Cheng Kung University, ROC. These procedures were approved by the Institutional Animal Care and Use Committee (IACUC) for the Use of Animal Subjects of National Cheng Kung University, ROC. The 3R animal use policy (Reduce, Replace and Refine) was reviewed and followed by the guidance from Ministry of Science and Technology, ROC.

The zebrafish embryos were incubated in chloroquine (CQ, Sigma, St. Louis, MO) which was dissolved in 0.3X Danieau zebrafish buffer with 0.003% 1-phenyl-2-thiourea (PTU) from dome stage (4.5 hour post fertilization, hpf). The CQ incubated embryos were collected at 32 hpf and fixed in 4% paraformaldehyde for overnight at 4 °C and then dehydrated in methanol. The dehydrated embryos were then performing whole mount *in situ* hybridization with different genes.

### Whole mount *in situ* hybridization (WISH)

The whole mount *in situ* hybridization in zebrafish embryo with *ff1b* ^26, 38^ or *star* (BioCat GmbH) was done as described before^39, 40^. Briefly, the embryos were fixed with 4% paraformaldehyde in PBS for overnight at 4 °C and then dehydrated by 100% methanol and stored at −20 °C. Before hybridization with probes, the dehydrated embryos were rehydrated with successive dilution of methanol by PBS, washed 5 times with PBST(PBS with 0.1% tween-20), digestion with proteinase K (10 μg/mL) for 2 minutes, re-fixed with 4% paraformaldehyde and washed 5 times with PBST. After rehydration and digestion, the embryos were incubated in prehybridization buffer for 2 hours and then transferred into hybridization buffer with antisense RNA probe at 70 °C for overnight. The hybridized embryos were washed with successive dilution of 0.2xSSC in PBST and stayed at 70 °C for 1 hour. The washed embryos were transferred into successive dilution of 0.2x SSC in PBST and finally incubated in blocking buffer with anti-dig antibody (1:10,000) for overnight at 4 °C. The antibody stained embryos were washed with 1XPBST for 6 time and then transferred into alkaline Tris buffer for 3 times. The embryos were stained with labeling mix contained Nitro Blue Tetrazolium (NBT) and 5-Bromo 4-Chloro 3-indolyl Phosphate (BCIP). After staining, the reaction was ceased by stop solution (1XPBS with 1 mM EDTA, pH 5.5). All the CQ incubated and *ff1b* mRNA injected experiments were performed 3 times (N = 3) independently. In each experiment, at least 30 embryos (n) were used in each different groups. The signal less than 50% compared with control group was defined as reduced signal in the interrenal gland tissue and the quantitative results were calculated by double blind test.

### Antibodies

The following antibodies were obtained commercially: anti-γ-tubulin (T6557), anti-cyclin A (C4710), and anti-α-tubulin (T9026) (Sigma, St. Louis, MO), anti-CDK2 (#2546), anti-CDK2 phospho-Thr160 (#2561), anti-Cleaved Caspase-3 (Asp175) (#9661), anti-CYP11A1 (D8F4F) (#14217), anti-Ad4BP/SF-1 (STF-1, D1Z2A) (#12800), and anti-LC3A/B (#12741) (Cell Signaling, Beverly, MA), anti-H2AX phospho-Ser139 (ab2893, Abcam, Cambridge, UK), anti- Beclin 1/ATG6 (NB500-249) (Novus, Littleton, CO), anti-cyclin E1 (HE-12, GTX23927), anti-actin (GTX109639) and anti-GAPDH (GTX100118) (Genetex, Irvine, CA).

### Plasmids, Luciferase reporter assay, and RNAi

Mammalian expression plasmids for mouse Ad4BP/SF-1 were constructed by subcloning the cDNA provided by Dr. Keith Parker (University of Texas Southwestern Medical Center, Dallas, TX) into pCMX^41^. pGL3-mCyclin E1-Luc reporter vector have mouse cyclin E1 promoter, located 5′ upstream of the lucifrerase gene provided by Dr. Johan Auwerx (Université Louis Pasteur, Illkirch, France)^25^. The luciferase reporter plasmids, Ad4 × 3, have three Ad4 sites, located 5′ upstream of the tk basal promoter. HEK293 or Y1 cells were seeded in 24-well dishes and transfected using Lipofectamine2000 (Invitrogen, Carlsbad, CA) with 200 ng of luciferase reporter plasmid (pGL3-mCyclin E1-Luc and pAd4 × 3-Luc reporter vector), 100 ng of pCMX-ß-gal, and control or Ad4BP/SF-1 siRNA oligonucleotides (100 nM). The cells were harvested 48 h after transfection, and the cell lysates were subjected to luciferase and ß-galactosidase assays as described previously. Ad4BP/SF-1 and Beclin 1 of adrenocortical Y1 and progenitor Leydig TM3 cell lines were depleted using annealed siRNA with the target sequence:

siAd4BP #1: 5′-AUGUGUGACAGCAUAGACUGCCACC [dt] [dt]-3′

siAd4BP #2: 5′-AUAAAGGUCUGGUCGGCCAUUCUGC [dt] [dt]-3′

siBeclin1: 5′-GAGUUGCCGUUAUACUGUU [dt] [dt]-3′ (Ambion, Life Technologies, Grand Island, NY).

Scrambled siRNA with the target sequence: 5′-GAUCAUACGUGCGAUCAGA [dt] [dt]-3′ was purchased from Sigma (Sigma, St. Louis, MO).

For siRNA transfection, 10 μl of Lipofectamine 2000 (Invitrogen, Carlsbad, CA) were mixed first with 500 μl Opti-MEM medium (Life Technologies, Grand Island, NY) for 5 min, then with 2 μl siRNA (100 μM) in 500 μl Opti-MEM medium, incubated at room temperature for 20 min before the mixture was layered onto cells in 1 ml DMEM/F12 medium (100 nM working concentration). Cells were harvested for further experiments 72 h after transfection.

A lentiviral system for Atg7 and Ad4BP gene silencing was obtained from the National RNAi Core Facility (Institute of Molecular Biology, Academia Sinica, Taipei, Taiwan). Short hairpin RNA (shRNA)-encoding pLKO.1 vectors were as follows:

pLKO.1-shluc (target sequence: 5′-CCTAAGGTTAAGTCGCCCTCG-3′)

pLKO.1-shsf1#2 (target sequence: 5′-CGCACCATCAAGTCTGAGTAT-3′)

pLKO.1-shsf1#3 (target sequence: 5′-CGTCTGTCTCAAGTTCCTCAT-3′)

Lentiviruses were produced by cotransfection with *pLKO*.*1*, *pCMVdelR8*.*91* and *pMD*.*G* for shRNA production in 293FT cells (Invitrogen, Carlsbad, CA) according to the protocols provided by the Taiwan National RNAi Core Facility.

### Chromatin immunoprecipitation (ChIP)

ChIP assays were performed basically with the procedure described by Winnay *et al*.^42, 43^. In brief, after cross-linking Y1 cells with 1% formaldehyde for 10 min at room temperature, they were rinsed twice with cold PBS, harvested in lysis buffer containing 20 mM Tris-HCl (pH 8.0), 10 mM EDTA 1% SDS, and incubated for 5 min at 4 °C. Next, their chromatin was sonicated to generate DNA fragments of 500–1000 bp. To reduce nonspecific background, the samples were precleared with normal rabbit IgG (1 μg/mL), salmon sperm DNA (100 mg/mL), BSA (10 mg/mL), and protein A agarose (50% slurry in PBS) for 60 min at 4 °C. After centrifugation at 1,000× g for 20 min, the supernatants were incubated with normal rabbit IgG or anti-Ad4BP/SF-1^37^ overnight at 4 °C. Next, protein A beads adsorbed to the immunocomplexes were collected by centrifugation, washed with 50 mM Tris-HCl (pH 8.0), 5 mM EDTA, 150 mM NaCl, 0.5% Nonidet P-40, 0.1% SDS, and Complete Protease Inhibitor Cocktail (Roche), and then washed again with 10 mM Tris-HCl (pH 8.0), 1 mM EDTA, 250 mM LiCl, and 0.1% Nonidet P-40. The beads were further washed three times with 10 mM Tris-HCl (pH 8.0) and 1 mM EDTA, and finally the chromatin fragments were eluted from the beads with 1% SDS in 0.1 M NaHCO3. After the cross-linking was reverted by heating at 65 °C 8 hr, DNA fragments were recovered using the QIAquick PCR purification system (Qiagen). The purified DNAs were resuspended in 100 μL water and 5 μL aliquots were used for PCR. Presence of the *Ccne1* promoter region was determined by PCR with the appropriate primer sets, indicated below. The PCR was carried out for 30 cycles and the products were resolved on 2% agarose gels to visualize the SYBR Green staining.

Primer pairs used for ChIP assays were as follows:

CyclinE1-1: 5′ GGTGAGGGTCACTTCACGTT 3′

CyclinE1-2: 5′ CAGCAGCAGGTCCTTTAACC 3′

### Cycloheximide chase assay

The expression plasmid of 3xFLAG-SF-1 (2.5 μg) was transfected into Y1 cells. 16 h after transfection in the presence or absence of CQ (100 μM), cells were treated with 50 μg/ml cycloheximide for different time periods and then harvested by CelLytic^TM^ M cell lysis reagent (Sigma, St, Louis, MO) with cocktail protease inhibitors. Cell extract was cleared by centrifugation for 10 min at 13000 rpm and supernatant was analyzed by immunoblotting.

### Statistical analysis

All results are expressed as the mean +/− S.D. from at least three independent experiments, more than 100 cells were counted in each individual group. Differences between two groups were compared using unpaired two-tailed t-tests, for which a p-value of less than 0.05 was considered to be statistically significant.

## Electronic supplementary material

Supplementary Figures
