# Supplementary material for: Lysosomal activity maintains glycolysis and cyclin E1 expression by mediating Ad4BP/SF-1 stability for proper steroidogenic cell growth
Source: Sci Rep. 2017 Mar 21;7:240. doi: 10.1038/s41598-017-00393-4 (PMC5428257; doi:10.1038/s41598-017-00393-4)
Supplement: Supplementary file 1 — Supplementary Figures [file 41598_2017_393_MOESM1_ESM.pdf]

Lysosomal activity maintains glycolysis and cyclin E1 expression by mediating Ad4BP/SF-1 stability for proper steroidogenic cell growth

Jhih-Siang Syu[1,2], Takashi Baba[3,4,a], Jyun-Yuan Huang[5,a], Hidesato Ogawa[6,a], Chi-Han Hsieh[7,8], Jin-Xian Hu[7,8], Ting-Yu Chen[1,2],Tzu-Chien Lin[1], Megumi Tsuchiya[9], Ken-Ichirou Morohashi[3,4], Bu-Miin Huang[1,10] *, Fu-I Lu[7,8] * and Chia-Yih Wang[1,2] *

1. Department of Cell Biology and Anatomy, College of Medicine, National Cheng Kung University, Tainan, 701, Taiwan.
2. Institute of Basic Medical Sciences, College of Medicine, National Cheng Kung University, Tainan, 701, Taiwan.
3. Division of Molecular Life Science, Graduate School of Systems Life Science, Kyushu University, Maidashi 3-1-1, Higashi-ku, Fukuoka 812-8582, Japan.
4. Department of Molecular Biology, Graduate School of Medical Sciences, Kyushu University, Maidashi 3-1-1, Higashi-ku, Fukuoka 812-8582, Japan.
5. Division of Genetics, Department of Obstetrics and Gynecology, National Cheng Kung University Hospital, Tainan, 704, Taiwan.
6. Advanced ICT Research Institute Kobe, National Institute of Information and Communications Technology, Iwaoka 588-2, Nishi-ku, Kobe 651-2492, Japan.
7. Department of Biotechnology and Bioindustry Sciences, College of Bioscience and Biotechnology, National Cheng Kung University, Tainan, 701, Taiwan.
8. Institute of Biotechnology, College of Bioscience and Biotechnology, National Cheng Kung University, Tainan, 701, Taiwan.
9. Graduate School of Frontier Biosciences, Osaka University, 1-3 Yamadaoka, Suita 565-0871, Japan
10. Department of Medical Research, China Medical University Hospital, China Medical University, Taichung, 404, Taiwan.

* Correspondence:

Chia-Yih Wang
phone: +886-6-2353535 ext. 5338
FAX: +886-6-2093007
email address: b89609046@gmail.com
Fu-I Lu
phone: +886-6-2757575 Ext.65620-612
FAX: +886-6-2766505
email address: fuilu@mail.ncku.edu.tw
Bu-Miin Huang
phone: +886-6-2353535 ext. 5337
FAX: +886-6-2093007
email address: bumiin@mail.ncku.edu.tw

a: These authors contribute to this study equally.

Supplementary methods

Immunofluorescence microscopy

Cells were grown on glass cover slips at 37°C before fixation with ice-cold methanol at -20°C for 6 min. After blocking with 5% BSA for 1 h, cells were incubated with antibodies for 24 h at 4°C. After extensive washing with PBS, cells were incubated with fluorescein isothiocyanate-conjugated and Cy3-conjugated secondary antibodies (Invitrogen, Carlsbad, CA) for 1 h in the dark. The nuclei were stained with 4',6-diamidino-2-phenylindole (DAPI, 0.1 μg/ml) simultaneously. After extensive washing, the cover slips were mounted in 50% glycerol on glass slides. Fluorescent cells were examined with an AxioImager M2 fluorescence microscope (Zeiss, Switzerland). The number of centrosomes from more than 100 cells was counted under the microscope in three independent experiments and shown as mean±SD. Student's t test was performed to analyze the difference between different groups.

Cell growth and MTT assays

After seeding overnight, cells were treated with different drugs for the indicated time periods then were trypsinzed and resuspended in PBS for cell number counting. For MTT (methylthiazole tetrazolium) assay, following drug

treatment, cells were washed with PBS followed by adding 1 ml MTT solution (2 mg/ml in PBS) in each well. After incubation for 3 h at 37°C, 2 ml DMSO was added and incubation in the dark for additional 30 mins. Absorbance was measured at the wavelength of 570 nm.

EdU incorporation assay

For EdU incorporation assay, EdU positive cells were stained by detecting fluorescence EdU signaling according to manufacturer's instruction (Invitrogen, Carlsbad, CA). The coverslips were then incubated with 1μg/ml DAPI for 20 min, and fluorescently stained nuclei were examined under a Axioplan2 microscope equipped with Plan-NEOFLUAR 20 x/0.50 NA objectives (Zeiss, Oberkochen, Germany) using two excitation filters: Filter 02 to visualize DAPI-stained nuclei and Filter 15 (Cy3) to visualize the EdU-labeled nuclei. Images were photographed with a DP70 digital camera (OLYMPUS) and were processed with Adobe Photoshop. The 400-500 nuclei per coverslip were blindly counted. The raw data of independent experiments were pooled to derive a final value of the percent labeled nuclei under each condition.

Cell cycle analysis

The cell cycle profile was analyzed by fluorescence-activated cell sorting (FACS) according to published method [1]. Briefly, cells were collected by trypsinization and re-suspended with PBS. Following centrifugation at 1,000 rpm for 5 min, cells were re-suspended with PBS-E (1 mM EDTA in PBS). After centrifugation, the pellet was re-suspended with 0.5 ml PBS-E and fixed with ice-cold 70% ethanol overnight at 4°C. Fixed cells were washed with PBS-E and stained with propidium iodide (PI, SouthernBiotech, Birmingham, AL) at room temperature for 1 h. DNA content of PI stained cells was measured by FACScan (Becton-Dickinson, San Diego, CA) and further analyzed by Kaluza software (Beckman Coulter, Brea, CA).

1 Chen, T. Y. *et al.* Cell Cycle-Dependent Localization of Dynactin Subunit p150(glued) at Centrosome. *J. Cell. Biochem.* **116**, 2049-2060 (2015).

Supplementary figures

Supplementary figure 1.

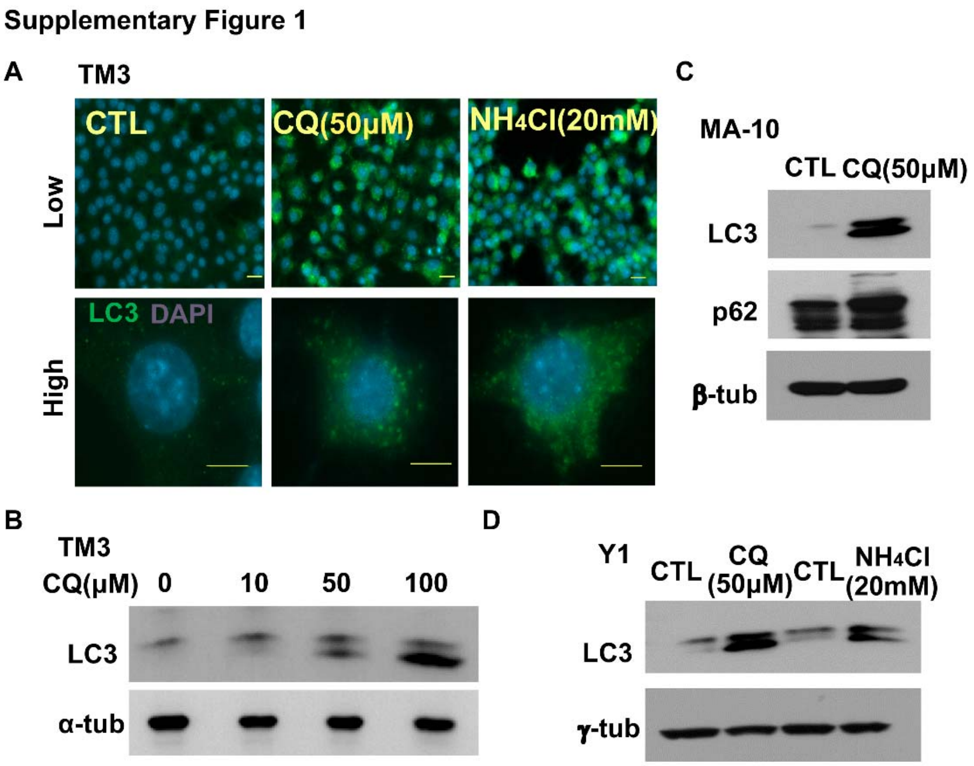

Supplementary figure 1. Lysosomal inhibitors induces accumulation of LC3 and p62.

(A-D) Treatment of cells with lysosomal inhibitors leads to accumulation of LC3 and p62. (A) Immunostaining of TM3 cells treated with CQ (50 μM) and NH_4Cl (20 mM) with antibody against LC3. Low: lower magnification; High: higher magnification. (B-D) Whole cell extracts of CQ (50 μM) and NH_4Cl (20 mM) treated TM3 (B), MA-10 (C), and Y1 (D) were analyzed by immunoblot with antibodies against LC3, p62, α-tubulin (α-tub), β-tubulin (β-tub), and γ-tubulin (γ-tub).

Supplementary figure 2.

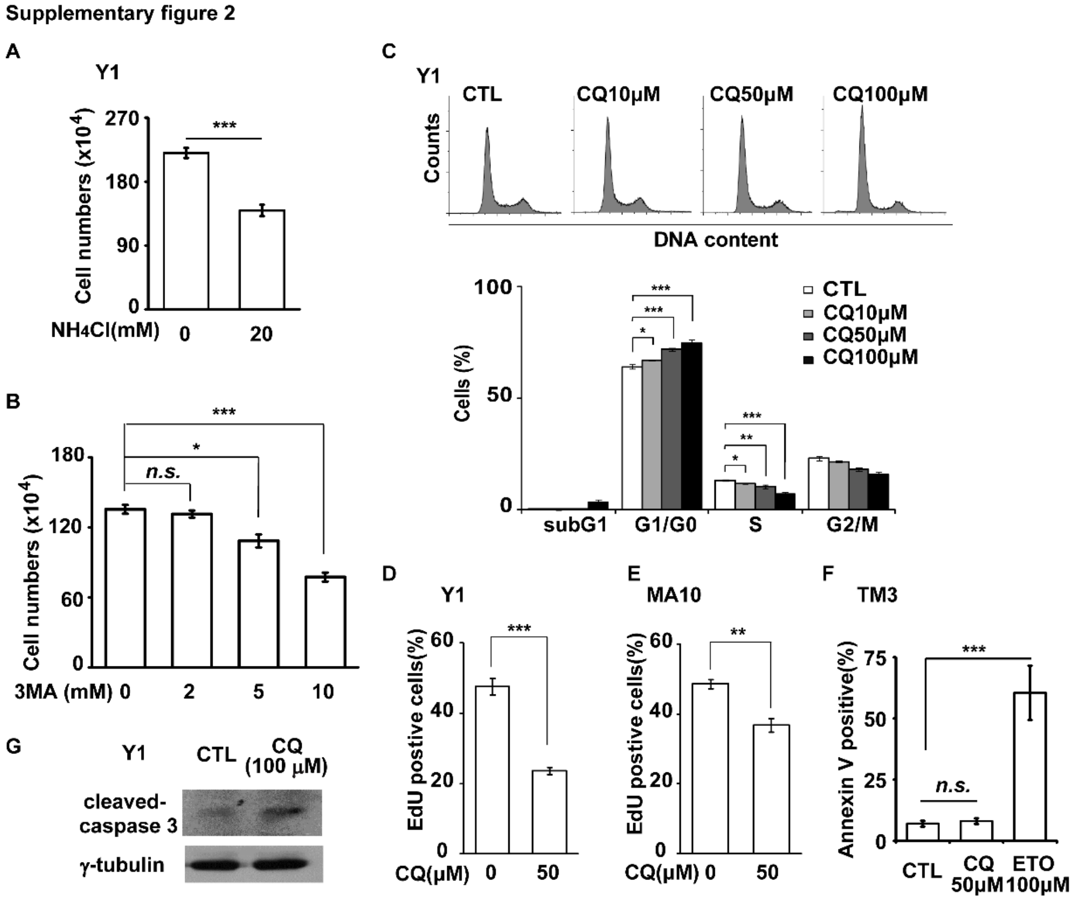

Supplementary figure 2. Inhibition of lysosomes leads to G1 arrest.
(A) Treatment of NH_4Cl for 24 h reduces the growth of Y1 cells. (B) High concentration of 3MA treatment reduced Y1 cell numbers. (C) CQ treatment (10, 50, or 100 μM, 24 h) leads to reduced S phase entry. Quantification of different cell cycle stages in Y1 cells in the presence or absence of CQ. (D-E) EdU incorporation is reduced in CQ-treated Y1 (D) and MA10 (E) cell lines. These results are mean +/- SD from three independent experiments; more than 1000 cells were counted in each individual group. (F) Low concentration CQ treatment (50 μM, 24 h) does not induces apoptosis. Quantitation results of Annexin V positive cells in the presence of CQ or etoposide (ETO, 100 μM, 24 h; positive control). (G) Whole cell extracts of CQ-treated Y1 cells at high concentration (100 μM) are analyzed by immunoblot with antibodies against cleaved-caspase 3 and γ-tubulin. n.s.: no significance; *: $P< 0.05$; **: $P< 0.01$; ***: $P< 0.001$.

Supplementary figure 3.

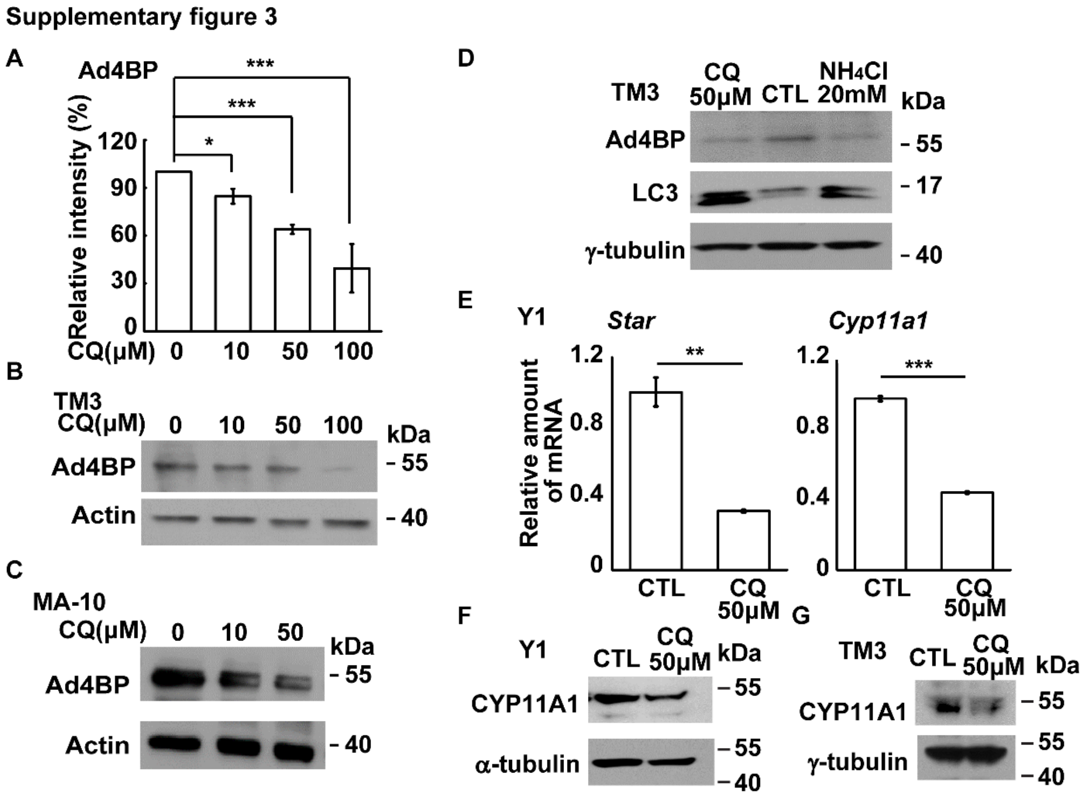

Supplementary figure 3. Lysosomal inhibition reduces Ad4BP/SF-1 and its downstream target gene expressions.

(A) Inhibition of lysosomes reduces the level of Ad4BP/SF-1.Quantitation of relative intensity of Ad4BP in (Fig. 4A). (B-D) Inhibition of lysosomes reduces the level of Ad4BP/SF-1. Whole cell extracts of CQ- or NH_4Cl-treated TM3 (B and D) or MA-10 (C) cell lines were analyzed by immunoblot with antibodies against Ad4BP/SF-1(Ad4BP), LC3, or γ-tubulin. (E-G) CQ (50 μM)-treatment reduces the expression of *Star* and *Cyp11a1* genes. (E) Quantitation of relative mRNA level in Y1 cells treated with CQ (50 μM). (F-G) Whole cell extracts of CQ (50 μM)-treated Y1 (F) or TM3 (G) cell lines were analyzed by immunoblot with antibodies against CYP11A1, γ-tubulin, and α-tubulin. *: $P< 0.05$; **: $P< 0.01$; ***: $P< 0.001$.

Supplementary figure 4.

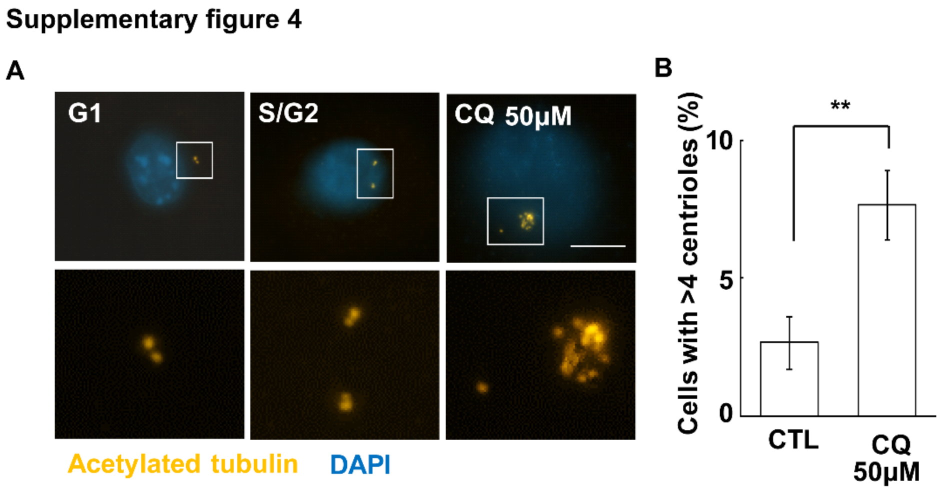

Supplementary figure 4. Lysosomal inhibition induces multiple centrioles in TM3 cells.

(A-B) CQ (50 μM) treatment induces centriole amplification. (A) Immunostaining of centrioles with antibody against acetylated tubulin. DNA is stained by DAPI. Scale bar is 5 μm. (B) Quantitation of cells containing multiple centrioles in the presence or absence of CQ (50 μM). All results are expressed as the mean+/-S.D. from at least three independent experiments. **: P< 0.01.

Supplementary figure 5.

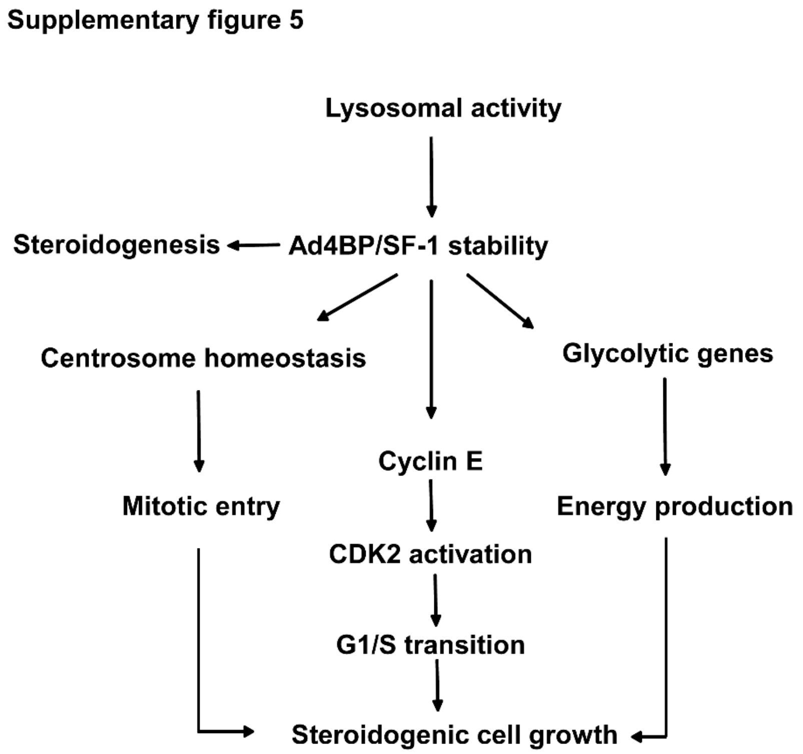

Supplementary figure 5. A flow chart depicting lysosomal activity action that maintains steroidogenic cell growth.

In steroidogenic cells, lysosomal activity maintains Ad4BP/SF-1 protein stability. Ad4BP/SF-1 regulates steroidogenesis. In addition, it also controls centrosome homeostasis, glycolysis, and cyclin E expression, thus in turn regulating steroidogenic cell growth.
